# Advice to Medical Students on Their Arrival in London

**Published:** 1816-09

**Authors:** 


*For the London Medical and Physical Journal.*

### *Advice to Medical Students on their Arrival in London.*

AS the period is arriving when the medical campaign, as it is usually termed, will open in London, permit one who has passed through the field to offer a few lines of advice to the new recruit.

Most young men bring with them to the metropolis a letter to some teacher, or to the physician or surgeon of some hospital. This they are eager to deliver as soon as possible, and never fail to meet with an agreeable reception. The first advice he receives is to become a pupil of the same hospital, and also of the anatomical teacher. All this is very well, as he must enter at some hospital, and the admission into either gives him access to all the others. He must also learn anatomy; and, though the largest hospitals are not always the best schools, yet, with great diligence, he may acquire a knowledge of dissection at either.

The more important concern is the medical teacher under whom he enters. This was once thought a matter of less consequence, because the more showy parts of surgery, particularly the operations, were considered as the best recommendations. But the examination at the Apothecaries' Hall has raised the medical branch of education to its proper rank. It is, indeed, by far the most important for the general ral practitioner and for the public, because it occurs the oftenest, and, excepting in accidents, which most surgeons acquire a knowledge in the management of, demands the most speedy assistance, whilst the success of a young practitioner in a chronic case which has baffled his predecessors, often stamps his reputation for ever.

The great advantages which the London school affords in this respect are almost incalculable. A professor is not forced on the pupil, who, like the late Dr. Cullen, may give half a course of medical lectures in the season without attending, or paying for which, the academic discipline was incomplete; but each young man may chuse his own teacher, and, what is more to the purpose, as each teacher gives two courses in the season, the student may have the advantage of learning the opinions, and benefiting by the experience, of more than one.

On this account, I would advise no young man to enter suddenly. Even if he has fixed on his hospital and his anatomical school, let him rest before he hastily becomes a perpetual pupil to any course of medicine. If he means to be in town more than a single half year, it will be adviseable for him to give up the thoughts of attending a medical course, at least till after the Christmas vacation. By this time he will see his way, and not have to regret that he has paid his money and cannot now retract. If he remains in town during the summer, it may be adviseable to attend a summer course. But, if his residence should be, from the necessity of his finances, only a single winter, he should certainly enter only a single course of medicine, and attend a different teacher for his second course. By these means he will not only learn more medicine, but will be master of opinions he may otherwise, from the partiality of the various schools, have been taught, without any just reason, to undervalue.

If his stay is long enough, I would even advise him to attend at least three different teachers in medicine; and, if the hospital dissecting room should be too full, to attend a course of anatomy during the summer months.

Some teachers have thought it necessary to add materia medica as a separate course; but this seems to be an unnecessary addition to an expensive education. The Apothecaries' Company, in their examination on this branch, require nothing more than what every apprentice must have learned, and what every course of pharmaceutic chemistry should contain.

